# Medical Teaching

**Published:** 1873-04-15

**Authors:** 


### Medical Examiner.

*A Semi-Monthly Journal of Medical Sciences.*

EDITED BY

N. S. DAVIS, M. D., AND F. H. DAVIS, M. D.

Chicago, April 15th, 1873.

### EDITORIAL.

### MEDICAL TEACHING.—CREDIT TO WHOM CREDIT IS DUE.

About two years since it was announced by the medical faculty of Harvard University, at Boston, that they had adopted the system of graded and consecutive teaching, similar to that pursued in all other departments of education, and should thereafter continue the medical instruction throughout the collegiate year, nine months. The announcement was very properly taken up and repeated with commendation by nearly all the medical periodicals in the country. That school was at once awarded the credit of having taken not only a very important step in advancing the cause of medical education, but also being the *first*, or "*pioneer*" institution in placing our professional education on a proper basis.

Although this sounded a little strangely in our ears, since the school with which we were connected had been successfully carrying out the same system, only more perfectly, for ten years, yet we were happy to have our friends in Boston receive all the commendation and encouragement they could get. But inasmuch as the Eastern journals continue pretty uniformly to award the credit of *pioneership* to the medical department of Harvard, we must gently remind them that there is a part of the United States of America west of the Alleghany mountains; and that the Chicago Medical College, now the medical department of the Northwestern University, was founded in 1859, expressly to test the feasibility of giving medical college instruction in the same methodical, consecutive manner as is pursued universally in other departments of education. It has steadily pursued its course, and since 1868 has rigidly graded its curriculum into three courses, corresponding with the three years of study, and all its students into their appropriate rank of junior, middle and senior classes. Its term of instruction continues nine months; the full winter term from the first of October to the middle of March, and the spring term from the first of April to the first of July, there being a vacation of one week from Christmas to New Year, and of two weeks in the last half of March. The institution has not only been sustained by the profession in the Northwest, but it has, to-day, a better college building, and is in immediate connection with a larger hospital than exists in Boston. Its laboratories for practical and analytical chemistry and microscopy, its museum of human and comparative anatomy, and the extent and variety of its clinical resources, are not excelled by any medical institution in this country. Will our Eastern friends inform us why they continue to award to the medical department of Harvard the credit of priority or leadership in the adoption of an improved system of medical instruction during the last two years, while the Chicago Medical College has carried out the same system, more perfectly, during the last fourteen years? We do not raise the question from any desire to depreciate the position of the Boston school. On the contrary, we rejoice that her faculty have had the courage to advance and plant their standard upon so good a foundation. And our experience has given us the utmost confidence that if they hold to their position firmly, they will not only succeed, but five years will not elapse before colleges in New York and Philadelphia, whose faculties are now mournfully predicting a failure of the enterprise, will be following their example.

